# Economic Costs and Benefits of a Community-Based Lymphedema Management Program for Lymphatic Filariasis in Odisha State, India

**DOI:** 10.4269/ajtmh.16-0286

**Published:** 2016-10-05

**Authors:** Eileen Stillwaggon, Larry Sawers, Jonathan Rout, David Addiss, LeAnne Fox

**Affiliations:** 1 Department of Economics, Gettysburg College, Gettysburg, Pennsylvania; 2 Department of Economics, American University, Washington, District of Columbia; 3 Church's Auxiliary for Social Action (CASA), Bhubaneswar, India; 4 Children Without Worms, Task Force for Global Health, Decatur, Georgia; 5 Division of Parasitic Diseases and Malaria, Centers for Disease Control and Prevention, Atlanta, Georgia

## Abstract

Lymphatic filariasis afflicts 68 million people in 73 countries, including 17 million persons living with chronic lymphedema. The Global Programme to Eliminate Lymphatic Filariasis aims to stop new infections and to provide care for persons already affected, but morbidity management programs have been initiated in only 24 endemic countries. We examine the economic costs and benefits of alleviating chronic lymphedema and its effects through a simple limb-care program. For Khurda District, Odisha State, India, we estimated lifetime medical costs and earnings losses due to chronic lymphedema and acute dermatolymphangioadenitis (ADLA) with and without a community-based limb-care program. The program would reduce economic costs of lymphedema and ADLA over 60 years by 55%. Savings of US$1,648 for each affected person in the workforce are equivalent to 1,258 days of labor. Per-person savings are more than 130 times the per-person cost of the program. Chronic lymphedema and ADLA impose a substantial physical and economic burden on the population in filariasis-endemic areas. Low-cost programs for lymphedema management based on limb washing and topical medication for infection are effective in reducing the number of ADLA episodes and stopping progression of disabling and disfiguring lymphedema. With reduced disability, people are able to work longer hours, more days per year, and in more strenuous, higher-paying jobs, resulting in an important economic benefit to themselves, their families, and their communities. Mitigating the severity of lymphedema and ADLA also reduces out-of-pocket medical expense.

## Introduction

Lymphatic filariasis (LF) afflicts an estimated 68 million people in 73 countries of Africa, Asia, Oceania, and the Americas^1^ and is one of the diseases targeted for elimination by the World Health Assembly (World Health Assembly Resolution WHA 50.29: Elimination of lymphatic filariasis as a public health problem. Fiftieth World Health Assembly, 5–14 May 1997, Resolutions and Decisions). The Global Programme to Eliminate Lymphatic Filariasis (GPELF) embodies two “pillars”: stopping new infections by the year 2020 and managing morbidity and preventing disability for persons already infected.^2^ Of the 73 endemic countries, 62 had initiated mass drug administration (MDA) for elimination of new infections as of 2014.^3^,^4^ As of 2015, 45 countries were considered “on track” to achieve elimination targets by 2020.^5^

An estimated 36 million people live with the disabling effects of LF, including about 17 million persons with chronic lymphedema, primarily of the legs, and also of the arms, breasts, and scrotum, and 19 million men with hydrocele.^1^ The remaining LF-infected persons are at risk of developing lymphedema or hydrocele. Programs to manage morbidity and prevent disability among infected persons, the second pillar of the GPELF, had been initiated in only 24 of the 73 endemic countries by 2014.^4^ This article examines the economic costs and benefits of one such program of morbidity management and disability prevention (MMDP) for alleviating the causes and effects of chronic lymphedema. Interventions for hydrocele differ from those for lymphedema and are not included in this study.

### Nature of the disease.

Larval forms of *Wuchereria bancrofti*, *Brugia malayi*, and *Brugia timori* are transmitted to humans by different species of mosquitoes, depending on world region. Lymphatic vessels are damaged by the presence of adult worms, causing lymphedema that worsens with age. Lymphedema is generally considered an adult condition, but damage to lymph vessels from filarial infection can begin in childhood.^6^,^7^ The progressive worsening of lymphedema is not inevitable; rather it is accelerated by recurrent episodes of acute dermatolymphangioadenitis (ADLA), disabling bouts of fever and intense pain lasting several days that are caused by bacterial infections.^8^,^9^ These infections generally enter the lower limbs where the skin is damaged by wounds or interdigital fungal infections.^8^,^10^

Each episode of ADLA further damages the lymph system and contributes to progression of chronic lymphedema, the severity of which has been classified into seven stages by Dreyer and others.^8^,^10^ In stage 1, lymphedema is generally relieved by limb elevation overnight. By stage 7, lymphedema is characterized by deep skin folds, knobs or protrusions, mossy lesions, interdigital lesions, and bad odor; the lymphedema usually extends above the knee and prevents activities of daily living.^8^,^10^ Worsening lymphedema increases vulnerability to entry lesions that lead to ADLA, which in turn worsens lymphedema. Several studies confirm the higher incidence of ADLA at higher stages of lymphedema,^11^–^18^ but others do not.^19^–^21^ Nevertheless, “[t]he epidemiologic association between ADLA frequency and stage, as well as extensive clinical experience from both filariasis-endemic and non-endemic areas, strongly suggest that ADLA episodes are a major—likely the most important—factor in lymphedema progression, particularly in filariasis-endemic areas.”^11^

### Prevention of increasing disability.

With recognition of the causes of ADLA and their role in progressive worsening of lymphedema came the realization that very simple and low-cost methods could prevent recurrent ADLA episodes and thus lymphedema progression. Washing the legs and feet with soap and clean water, drying the limbs with clean towels, applying antifungal creams or antibiotic ointments to interdigital lesions, elevating affected limbs, exercising to improve lymphatic and venous drainage, and wearing shoes have been shown to be effective in reducing the number of episodes of ADLA^12^,^15^,^17^,^22^–^34^ (see Supplemental Information for additional discussion). Several studies have also found that limb hygiene was associated with reduced leg volume and regression in lymphedema stage.^25^,^31^,^35^

Very simple interventions can have a substantial impact on quality of life. Each episode of ADLA can mean several days of excruciating pain. Lymphedema limits mobility and daily activities; it can be disfiguring and lead to stigma. Reducing ADLA and lymphedema allows LF-affected persons to engage more easily in family and community life as well as employment.

### Previous studies of the economic cost of LF.

Numerous studies have described the economic cost imposed by ADLA or lymphedema. When people seek medical attention for their chronic lymphedema or during an ADLA episode they incur out-of-pocket expenses for consultations, tests, medications, and transportation costs. The quantitatively more important components of the lifetime economic cost of LF are the lost earnings in paid employment and loss of unpaid household labor. Those with lymphedema or ADLA may be forced to work fewer days per year or fewer hours per day and may earn a lower wage because they cannot engage in strenuous labor.

Chu and others^36^ estimated the benefits of MDA from the first 8 years of the GPELF. They included patient out-of-pocket costs of medical consultations, medications and travel, lost wages due to reduced hours of work or days lost to ADLA, and medical costs to the public sector. They found that the economic cost of LF could be reduced by at least US$20 billion by preventing transmission of filarial infection.

Two studies in Odisha State (formerly Orissa), India, found that the average annual out-of-pocket cost for medical care for lymphedema and ADLA was about US$14,^37^,^38^ more than 10 times the average daily wage of unskilled rural workers in the state.^39^ Studies elsewhere in India and in other countries also found substantial out-of-pocket costs for medical care for lymphedema and ADLA.^40^–^44^

Each ADLA episode leads to a loss of 3–12 days of work, with an average reported in India of more than 4 days; annual incidence of ADLA varies widely among different studies in India, ranging from 1.6 to 7.6 episodes.^11^,^15^,^40^,^45^–^50^ Chronic lymphedema at advanced stages can be completely disabling and prevent wage employment or the performance of household tasks. Those at intermediate stages of lymphedema may have partial disability with substantial earnings loss. For example, daily output measured in yards of cloth was found to be 27% lower for weavers with lymphedema than for those without.^51^ Estimates of reduced time spent in paid or unpaid employment (measured in either hours per day or days per year) for those with lymphedema range from 13.0% to 19.5%.^38^,^42^,^44^,^49^,^51^,^52^ Together with out-of-pocket medical costs, those earnings losses are an extraordinary economic burden on some of the poorest people in India.

## A Community-Based Program in Khurda District, Odisha State, India

The present work examines the economic costs and benefits of the lymphedema management program implemented by the Church's Auxiliary for Social Action (CASA), an Indian nongovernmental organization (NGO), with technical assistance provided by the U.S. Centers for Disease Control and Prevention. In 2005, 40 local NGOs conducted a house-to-house census of 1.3 million persons in the rural and peri-urban areas of Khurda District in Odisha State, an LF-endemic area, and identified all residents with lower-limb lymphedema, recording age, gender, lymphedema stage, and number of ADLA episodes in the previous year.^53^–^55^ From 2007 to 2010, CASA provided services to more than 21,000 persons identified in 1,447 villages in a community-based program utilizing community health workers to train LF patients in leg washing and use of topical antibiotic and antifungal treatments.^54^ In 2009 to 2011, 370 patients from villages not yet enrolled in the CASA program in Khurda were recruited in a prospective cohort study examining the effectiveness of the lymphedema management program. Participants reported a significant decrease in perceived disability after 2 years in the program, with greater improvements in patients with moderate or advanced lymphedema. Patients also reported losing 2.5 fewer work days per month after 1 year in the program.^56^

In another study of the 370 patients in the limb-care program, ADLA episodes decreased 34% over 24 months. The percentage of persons whose lymphedema progressed (worsened) decreased and the percentage of those whose lymphedema regressed (improved) increased. Use of soap was associated with decreased incidence of ADLA among persons without entry lesions.^35^ Per-person program costs were US$10.00 to US$12.50 for the 24 months. Based on 29 days of lost productivity per year recovered as a result of the limb-hygiene program, it was estimated that 1,600 person-years of labor were saved in the first year of the CASA program covering more than 21,000 people.^54^ Clinical data for this economic analysis are based on the 2005 census and the pilot studies of 2009–2011 mentioned above.

## Method

Although the individual experience of persons with lymphedema due to LF varies, there is a general tendency, in the absence of intervention, toward increasing stage of chronic lymphedema and increasing frequency and severity of ADLA episodes with age.^57^–^59^ The purpose of MMDP for people with lymphedema is to prevent ADLA and stop the progression of chronic lymphedema. We began by calculating the age distribution of chronic lymphedema and number of ADLA episodes per year for the population in the 2005 census of households in Khurda District mentioned above. We grouped people from 8 to 72 years of age into 5-year age cohorts and calculated the number of people at each stage of lymphedema and the number of ADLA episodes in the previous year for each age cohort. Using Microsoft Excel (Microsoft Corporation, Redmond, WA), we estimated the economic cost of morbidity and disability over the working lives of affected persons without lymphedema management and the projected reduction in those costs that would result from implementation of a community-based lymphedema management program.

### Age distribution of lymphedema stage and ADLA without and with lymphedema management.

We postulated two scenarios. In both scenarios, MDA has ended transmission of the LF parasite, but it has not reduced lymphedema or ADLA. Every 5 years, the oldest age cohort is retired from the population and younger cohorts move forward. Below age 8 years, no newly infected persons enter the treatment population because of the effects of five rounds of MDA in stopping transmission of filariasis.^1^

In the no-treatment scenario, there was no intervention to improve limb care, prevent ADLA, or slow progression of lymphedema. As each cohort ages, its average lymphedema stage and frequency of ADLA increase so that, 5 years from now, each group will have the morbidity distribution that its next older cohort has at present.

In the treatment scenario, we assumed that the community lymphedema management program on average halts the progression of lymphedema. In every age cohort, we assumed that progression of lymphedema stage for some is offset by regression for others, and thus each age cohort maintains the same distribution of lymphedema that it had at the beginning of the lymphedema management program. Based on the results of the limb-care program in Khurda, we assumed that the number of ADLA episodes for each age cohort will be one-third less than that in the no-treatment scenario.^35^

### Costs.

Using the two sets of morbidity distributions—ADLA and lymphedema stage with and without lymphedema management—we calculated the economic cost for each scenario. The difference between the two (the cost saving) is the economic benefit of lymphedema management.

Costs were calculated from the societal perspective, but we included only out-of-pocket costs to patients and their caregivers for clinic visits, medical tests, travel, and medications, and lost earnings for patients due to chronic lymphedema and ADLA. The earnings loss due to lymphedema and ADLA episodes arises from fewer work days, fewer hours per day, and/or lower daily wages. Based on costs reported in the literature, we calculated the total out-of-pocket costs and lost earnings with and without lymphedema management for each age cohort during the initial 5-year period, and then for every 5 years until the cohort ages out of the analysis at the age of 72 years. The economic benefit from the lymphedema management program is the difference between the two estimates of the total cost. We then compared the direct costs of implementing the program to the economic benefits of lymphedema management.

All costs were estimated in US dollars for 2008, discounting future costs at 3% per year. To determine what economists call the present discounted value, future costs and benefits are assumed to be worth less than current ones and are weighted less than those in the near term.^60^ Real wages (adjusted for inflation) and real expenditure on medical care were projected to rise 4% per year. Total cost was estimated over the working lives of all persons up to age 72. Table 1 lists the parameter values estimated for the calculation of lifetime out-of-pocket cost and earnings loss. (See Supplemental Information for explanation of data sources and derivations of parameter values for out-of-pocket medical costs, average number of days worked per year, lost work days due to chronic lymphedema and ADLA, wage rate, and the rate of increase in real wages and in the real cost of medical care.)

We used conservative estimates for improvement in ADLA, stage progression, and lost work days and hours. Predictions for real wage growth and the cost of medical care over the next 60 years are subject to considerable uncertainty. Thus, we performed sensitivity analysis using higher estimates of lost work days and lower and higher estimates of rate of growth of real wages and costs, the results of which are reported in the Supplemental Information.

## Results

We found progression of lymphedema with age in Khurda District as found in other studies.^57^–^59^ Table 2 shows the distribution of lymphedema stage by age, grouped in 5-year cohorts. From the youngest to the oldest, there is a steady decrease in the proportion of people in stage 1. For higher stages, we found the opposite trend. The average lymphedema stage rises monotonically with age (from 1.20 in the youngest cohort to 2.35 for people in their 70s.). Table 3 shows the percentage of persons in each lymphedema stage experiencing 0, 1, 2, and 3 ADLA episodes in the previous year. Our analysis of the Khurda data confirmed, as some studies have found^11^,^13^–^18^,^48^,^57^ but others have not,^19^–^21^,^58^ that those in higher stages of lymphedema are likely to have more ADLA episodes.

### Economic cost with and without lymphedema management.

Days of work lost due to chronic lymphedema and to ADLA episodes for each age cohort without an intervention are shown in the second and third columns of Table 4. We calculated the lost earnings from partial or total disability as the total number of work days lost times the average wage for rural households in Odisha State. Derivation of work days lost and the wage is described in the Supplemental Information.

Current out-of-pocket spending for medical attention for lymphedema and for ADLA episodes for each age cohort is shown in the fifth and sixth columns of Table 4.

We calculated the economic cost of lymphedema and ADLA in two scenarios, with and without a community-based lymphedema management program. Without the program, each cohort would progress through lymphedema stages as had older cohorts and more people would experience episodes of ADLA, replicating the experience of older cohorts. The total economic cost of lymphedema and ADLA is calculated as the present discounted value of the sum of out-of-pocket costs and lost earnings over the working lives of all persons with morbidity identified in Khurda. For this population, the total lifetime economic cost without lymphedema management is US$47.4 million.

We then calculated the economic cost for this population in a scenario with community-based lymphedema management; people on average remain in the same stage of lymphedema over time and experience on average one-third fewer ADLA episodes per year than they would have without the limb-care program. This scenario, based on the results of the Khurda limb-care program, represents a substantial gain in quality of life for more than 17,000 people who can expect a reduction in number of episodes of ADLA and stabilization of lymphedema stage or possible improvement. The present value of the total economic cost for this population after lymphedema management is US$21.3 million.

The present value of the benefit of lymphedema management (the reduction in economic cost) for this population is US$26.1 million, or US$1,648 per participant of working age. When the community-based lymphedema management program was implemented in Odisha, the average daily wage for low-skilled agricultural workers in the state was US$1.31.^39^ Thus, the present value of per-person economic benefit from the limb-care program was equivalent to 1,258 days of earnings. To implement and operate the Khurda community-based lymphedema management program for 2 years cost between US$10.00 and US$12.50 per person.^35^ The average participant in the program can expect lifetime economic benefits that are between 132 and 165 times the per-person cost of the program. The results are robust to changes in parameters for wage and price increases and work days lost (see Supplemental Information for sensitivity tests of our assumptions about parameter values).

## Discussion

Lymphedema and episodes of ADLA in filariasis-endemic areas diminish the quality of life of affected persons due to pain, stigma, numerous days of illness each year, restricted mobility, and reduced participation in family and community life. They also impose a substantial economic cost on affected persons and their families and diminish the potential economic strength of communities. Programs to provide care for persons with lymphedema and ADLA (as well as hydrocele) in filariasis-endemic areas are mandated by the GPELF. Beyond the ethical mandate to improve quality of life for affected persons, there are strong economic arguments for investing in the care of persons affected by filariasis, which the results of this research confirm. With adequate limb care, patients are better able to support themselves and provide for their families. Children and other dependents of affected persons could have greater access to better nutrition and the opportunity to attend school if the wage earner is healthier. Family members are relieved of the burden of caring for persons who are bedridden due to ADLA or advanced lymphedema and can contribute better to household income and domestic tasks. The community's economy is strengthened with fewer of its members disabled by lymphedema and ADLA and fewer of its families in poverty.

### Extent of the problem.

Our dataset was based on a morbidity census that was taken by visiting every household in the rural and peri-urban areas of Khurda District and found more than 17,000 persons with some degree of lower-limb lymphedema, 1.3% of the regional population.^55^ Two-thirds of persons with lymphedema, however, were in stage 1 or 2. These results suggest the possible invisibility of persons in other locations who have subclinical lymphatic damage due to LF infection, early-stage lymphedema, or infrequent episodes of ADLA and who remain at risk for worsening ADLA, advanced lymphedema, and disability. Where morbidity estimates are based not on a census, but on the number of people who seek treatment of chronic lymphedema, ADLA, or hydrocele, prevalence could be greatly underestimated.

Another issue highlighted by the Khurda census data is the long time horizon of lymphedema and ADLA and their ongoing economic cost. Even if new infections are stopped by 2020, some people whose lymph vessels are already damaged will experience ADLA episodes and lymphedema for the rest of their lives. Lymphedema and ADLA can necessitate out-of-pocket medical costs and cause a loss of earnings from reduced hours, absenteeism, and reduced intensity of work for 60 years or more. Indeed, the younger the cohort, the greater are the economic losses that accrue over their working lives. Thus, it is of critical importance to begin lymphedema management as soon as possible and to include young people and others who may have subclinical lymphatic damage and few or no ADLA episodes. Very low-cost interventions initiated now can save a lifetime of suffering and lost earnings.

### Potential benefits nationally and internationally.

Implementation of lymphedema management throughout India would reap benefits many times greater than in Odisha alone, one of the poorest Indian states. In ranking 20 Indian states by the daily wage rate, Odisha is in the bottom quartile in nine of 10 unskilled rural occupations (Table 3a in Labour Bureau^39^). Even though LF generally affects the poorest people, in most other states, rural wages at all levels are higher than in Odisha. Consequently, the earnings loss of lymphedema and ADLA would be greater and the benefits of a lymphedema management program would also be greater in other Indian states than in Odisha. In other countries, community- and clinic-based limb-care programs have demonstrated the efficacy of low-cost interventions in reducing the number of ADLA episodes and stabilizing or improving lymphedema stage. It is reasonable to conclude that those improvements in quality of life would also yield economic benefits. Since the largest component of the cost of lymphedema and ADLA is the loss of wages—and the largest benefit is regained productivity—it is likely that gains elsewhere would be greater than in Odisha because it has lower wages than in most other LF-affected areas.

### A public health approach: integration with other programs.

Every filariasis-endemic country has numerous other serious health problems competing for scarce resources, whether from government sources or community NGOs. While some aspects of elimination programs may require a vertical, or disease-specific, approach, policymakers are finding that integration of control programs for multiple diseases can have logistical and economic advantages.

With morbidity management as well, there could be important advantages to integrated programs. Limb care in particular might be integrated across several diseases common in India and in other countries. India has the world's highest burden of Hansen's disease (leprosy),^68^ also present in several other LF-endemic countries, which can necessitate lifelong limb care. There are an estimated 4 million people globally with podoconiosis, for whom limb treatment is similar to that for LF.^69^ Diabetes, now common in affluent countries, is an increasing problem in low- and middle-income populations. Foot protection, wound care, and limb hygiene are all important for diabetes care as well. Providing education and support for people with limb-care needs can be carried out in the public sector or in NGO-run programs, whether at the health facility or community level, with the potential for substantial cost economies as well as social benefits. Integrated programs can help reduce the social isolation of disfiguring and debilitating diseases. The emphasis on rehabilitating people in traditionally marginalized groups, such as people with Hansen's disease and LF, and helping them maintain their work performance or return to participation in community life, carries an important message of inclusion.^70^

Programs to educate people in limb washing require access to clean water. Water, sanitation, and hygiene (WASH) programs are essential for limb care, as well as to reduce breeding grounds for species of mosquito vectors of LF that flourish in open sewers. Reduced costs for limb-care programs, as well as reduced disability for LF patients, are important externalities that should be included in estimations of the benefits of WASH programs.

### Limitations.

To model the economic impact over the lifetimes of those with lymphedema and ADLA, we have made conservative assumptions about labor markets, the impact of disability on productivity, length of working life, and other parameters. We assumed flexible labor markets that could absorb workers who are rehabilitated through lymphedema management programs without exerting downward pressure on wages. This is reasonable because, although LF morbidity is a serious problem in endemic areas, the number of people affected is still a small proportion of the available labor force. Increases in labor supply from reduced morbidity would have little effect on the labor market because the expected gains from lymphedema management programs extend over the working lives of cohorts, rather than acting as a shock to labor markets at a moment in time. Moreover, any increase in earnings in the wake of a lymphedema management program is likely to be spent in the local economy, which could stimulate job growth and offset any downward pressure on wages from increased labor supply. We have not included a local multiplier effect and therefore the economic benefit of the intervention over time is substantially underestimated.

While the present analysis shows substantial gains from a community-based lymphedema management program for the Khurda population—US$26.1 million or US$1,648 per person enrolled—we think that those figures underestimate the economic costs of untreated LF morbidity and the benefits of lymphedema management. Our baseline estimate for earnings loss due to chronic lymphedema and ADLA was below the range found in several other studies. We did not include lost work time for youths until they reach the 20-year cohort, although young people in poor rural areas are generally employed, even below the age (15 years) included in government employment statistics. In addition, we chose only low-wage occupations in rural areas (omitting semi-skilled trades with higher wages) to set the daily wage rate in our modeling. Finally, we assumed that a successful lymphedema management program would freeze the age structure of lymphedema. Recent studies, however, find that lymphedema management leads to net regression of lymphedema stage as well as reduction in the number of ADLA episodes.^12^,^15^,^17^,^23^–^35^,^56^

This study underestimates the costs of LF morbidity and the benefits of lymphedema management in other ways. We have attempted to measure only the economic costs that fall directly on persons with chronic lymphedema and ADLA in a filariasis-endemic area. We exclude costs to others, including society as a whole or government. Subsidized care in government-run clinics, for example, is ultimately financed by the taxpayer. Reducing disease progression and disability reduces the need for subsidized care in the future, a benefit to taxpayers that is not included in our analysis.

We do not include any accounting of other externalities of chronic lymphedema and ADLA. For example, we do not include the lost work time of family caregivers for those disabled from ADLA and lymphedema, nor the impact on child nutrition and schooling, which would affect the child's future earnings. Since we have not measured these second-order costs of morbidity and benefits of lymphedema management programs, our calculations substantially understate the reduction in the economic cost of lymphedema and ADLA that a lymphedema management program would generate.

Chronic lymphedema and episodes of ADLA impose a substantial physical burden on the population of Khurda District, a filariasis-endemic area, and that disease burden increases with age. The economic burden of lymphedema and ADLA is also substantial. A low-cost program of lymphedema management based on limb washing and topical medication for infection can reduce the economic burden on poor populations affected by filariasis morbidity by 55%. The net benefit per person over the lifetime is more than 130 times the per-person cost of the program and equivalent to more than 1,250 days of earnings for the average person affected by filariasis.

Programs for MMDP are mandated by the twin pillars of the GPELF. Low-cost interventions have been shown to be effective in reducing the frequency of episodes of ADLA and slowing progression of lymphedema. This study demonstrates that the economic benefits of such interventions far exceed the costs and result in very significant benefits to filariasis-affected people and their communities.

## Supplementary Material

Supplemental Datas.

## Figures and Tables

**Table 1:** Parameter values for medical costs and earnings loss due to lymphedema and ADLA*

Parameter	Baseline estimate 2008–2009	Source
Annual per-person out-of-pocket medical costs for chronic lymphedema	US$10.96	38
Per-episode out-of-pocket medical costs for ADLA	US$2.04	37
Annual increase in real cost of medical care for chronic lymphedema and ADLA	4%	61–66
Annual discount rate	3%	60
Average daily wage rate	US$1.31	39
Annual increase in real wages	4%	61–66
Lost work days per episode due to ADLA	4	37
Average number of days worked per year	289	67
Percentage of work days lost annually due to chronic lymphedema		
Stages 1–2	0	38,42,44,49,51,52
Stage 3	20	
Stage 4	50	
Stages 5–7	100	

ADLA = acute dermatolymphangioadenitis.

* Derivation of values is explained in Supplemental Information.

**Table 2:** Stage of lymphedema by age cohort in Khurda census, 2005

Age cohort (years)	Number of respondents	Percentage of age cohort at each stage of lymphedema								Average stage
		Stage of lymphedema								
		1	2	3	4	5	6	7	Total	
8–12	74	86.5	6.8	6.8	0.0	0.0	0.0	0.0	100.0	1.203
13–17	137	78.8	15.3	2.9	2.2	0.0	0.7	0.0	100.0	1.314
18–22	267	70.4	18.0	8.6	2.6	0.0	0.0	0.4	100.0	1.453
23–27	443	61.9	24.6	9.5	2.9	0.2	0.9	0.0	100.0	1.578
28–32	866	56.8	24.0	15.1	2.2	0.7	0.9	0.2	100.0	1.696
33–37	1,158	47.8	30.3	16.4	3.7	0.7	0.5	0.6	100.0	1.832
38–42	1,845	43.0	29.7	19.1	5.0	1.2	1.0	1.0	100.0	1.987
43–47	1,789	40.9	29.2	21.0	5.5	1.7	1.2	0.6	100.0	2.037
48–52	2,257	38.0	29.2	23.4	6.0	1.7	1.0	0.8	100.0	2.104
53–57	1,723	34.5	28.1	25.2	8.9	1.7	0.8	0.8	100.0	2.208
58–62	2,441	31.0	30.1	25.8	8.6	2.5	1.4	0.6	100.0	2.280
63–67	1,400	29.3	31.2	25.8	9.1	2.0	1.9	0.7	100.0	2.318
68–72	1,453	29.9	28.4	26.8	10.1	2.3	1.7	0.8	100.0	2.352
Total	15,853	39.5	28.6	21.9	6.6	1.6	1.1	0.7	100.0	2.084

**Table 3:** ADLA episodes in previous year experienced by persons in each lymphedema stage in Khurda census, 2005

Stage of lymphedema	Percentage of persons in each stage with ADLA episodes				
	0 episode	1 episode	2 episodes	3 episodes	Total
1	17.1	68.7	7.8	6.4	100.0
2	16.1	71.4	7.2	5.2	100.0
3	15.2	69.4	9.2	6.3	100.0
4	14.2	68.5	9.6	7.7	100.0
5	15.7	58.7	12.3	13.3	100.0
6	10.8	62.1	12.8	14.3	100.0
7	12.6	57.1	15.1	15.1	100.0
Average	16.1	69.3	8.2	6.4	100.0

ADLA = acute dermatolymphangioadenitis.

**Table 4:** Work days lost annually, annual earnings lost, and annual out-of-pocket medical costs due to lymphedema and ADLA for each age cohort at program start, 2008–2009*

5-year cohort	Work days lost annually		Annual earnings lost due to lymphedema and ADLA	Annual out-of-pocket medical costs	
	Due to lymphedema	Due to ADLA		Due to lymphedema	Due to ADLA
18–22	2,630	1,084	US$4,865	US$2,926	US$553
23–27	5,751	1,796	US$9,887	US$4,855	US$916
28–32	14,941	3,568	US$24,247	US$9,491	US$1,820
33–37	23,265	4,784	US$36,744	US$12,692	US$2,440
38–42	50,835	7,568	US$76,508	US$20,221	US$3,860
43–47	53,754	7,460	US$80,190	US$19,607	US$3,805
48–52	72,626	9,204	US$107,197	US$24,737	US$4,694
53–57	63,725	7,096	US$92,775	US$18,884	US$3,619
58–62	98,405	10,224	US$142,303	US$26,753	US$5,214
63–67	57,858	6,196	US$83,910	US$15,344	US$3,160
68–72	64,245	6,192	US$92,272	US$15,925	US$3,158

ADLA = acute dermatolymphangioadenitis.

* See Supplemental Information for derivation of values.
