# Supplementary material for: Economic Costs and Benefits of a Community-Based Lymphedema Management Program for Lymphatic Filariasis in Odisha State, India
Source: Am J Trop Med Hyg. 2016 Oct 5;95(4):877–84. doi: 10.4269/ajtmh.16-0286 (PMC5062793; doi:10.4269/ajtmh.16-0286)
Supplement: Supplementary file 1 [file SD6.pdf]

SUPPLEMENTAL INFORMATION

DETAILED METHODS AND SOURCES

This supplement provides detailed information on the sources used to determine parameter values and the method of calculating the results for our modeling of the economic cost of lymphedema and acute dermatolymphangioadenitis (ADLA) in an area with endemic lymphatic filariasis (LF).

Efficacy of lymphedema management. Numerous studies have provided evidence of the efficacy of simple programs of limb care in stopping or reversing progression of lymphedema and reducing the number of episodes of ADLA. Many successful lymphedema management programs have been community-based educational campaigns. The World Health Organization explained the rationale for community-based efforts, saying "the social model views disability as a matter of an individual's full integration into society and prescribes social action to make the environmental modifications necessary for the full participation of people with disabilities in all areas of social life."[1]

In Egypt, 2 years after instruction in limb washing and application of antifungals and antibacterial ointments to small wounds, participants reported a 57% reduction in ADLA episodes per year.[2] In Nigeria, under community-based care, 90% fewer patients experienced two episodes of ADLA per month after 12 months of treatment.[3] In Burkina Faso, after 4½ months of a leg-hygiene program, the percentage of patients experiencing an ADLA episode in the previous month was reduced by half.[4] A community program of home-based care in Sri Lanka found that the number of persons experiencing one or more ADLA episodes decreased by 64% (Table 3)[5] and 66% perceived reduced lymphedema after 1 year.[5]

Most community-based lymphedema management programs for filariasis reported in the literature have been in India. In Kerala, 1-day health camps to teach leg washing and care of bacterial entry points were followed up after 6 months by questionnaires on disease symptoms (redness, swelling, odor, wound, and fever) and quality of life. Almost all participants (96%) reported reduced symptoms after following the hygiene regimen.[6] Also in Kerala, placebo-controlled trials examined the efficacy of different treatments for reducing ADLA that included diethylcarbamazine (DEC) and either ivermectin or penicillin. Participants in all arms were enrolled in a comprehensive foot-care program.[7,8] Improved foot care was found to be an effective treatment even for those who received no medication. A follow-up study in which visits were carried out a year or more later, with no additional interventions, found a 72.5% reduction in the frequency of ADLA, confirming the lasting efficacy of education efforts to reduce morbidity.[9]

In Tamil Nadu, in a trial of three drugs to prevent ADLA, all arms of the trial practiced limb hygiene. Even the control group, practicing only leg washing, had reduced incidence of ADLA in the treatment year and beyond.[10] In two LF-endemic districts in Kerala and Karnataka States, patients with grade II and III lymphedema (of grades I–IV) were trained in limb hygiene in LF camps and evaluated 3 months later. In Kerala, 25% fewer patients had episodes of ADLA, and in Karnataka, 73% fewer patients had ADLA episodes[11] (see also Ref. 12).

In addition to community-based educational programs, a few lymphedema management programs have been based in clinics. In Haiti, one such program emphasizing self-care, especially washing of the legs, was associated with a 69% reduction in ADLA episodes for all participants and reduction in leg volume for participants with stage 2 lymphedema.[13] In another study in Haiti, it was found that washing affected legs with soap was associated with a reduction of ADLA episodes from 1.1 to 0.4 per person-year, a 64% reduction, over the 12-month study.[14] In contrast, a clinic-based program in Togo led to a small, but statistically insignificant, decline in the number of ADLA episodes and a statistically significant increase in the number of patients whose lymphedema prevented them from washing or getting out of bed.[15]

A recent systematic review and meta-analysis concluded that the "available evidence strongly supports the effectiveness of hygiene-based lymphedema management in LF-endemic areas" for the prevention of ADLA.[16] Their meta-analysis found that participation in such programs was associated with decreased percentage of patients reporting at least one episode of ADLA (odds ratio [OR] = 0.29, 95% confidence interval [CI] = 0.12–0.47) and lower incidence of ADLA (OR = 0.32, 95% CI = 0.25–0.40).[16]

Some studies suggest that mass drug administration (MDA) can reduce lymphedema and frequency of ADLA in persons already infected,[17–26] but other studies do not.[10,27–29] Consequently, available evidence does not warrant incorporating in the model any effect of MDA on lymphedema and ADLA.

The results of the Khurda community-based limb-care program are in line with the results discussed above. We based our parameters—halting progression of lymphedema and one-third reduction in ADLA episodes—on the literature above and the outcomes of the Khurda interventions.[30,31]

Clinical data. The objective of the study is to assess the economic effect of a morbidity management and disability prevention program that changes the age distribution of lymphedema and ADLA in a population over time. We derive the age distribution of morbidity from a census of households conducted in 2005 in Khurda District, Odisha State, India, by 40 Indian nongovernmental organizations. The census recorded age, gender, lymphedema stage, and number of ADLA episodes in the previous year. The data we used were stripped of personal identifiers and were thus anonymous. Consequently, no ethical clearance was warranted. We used Microsoft Excel (Microsoft Corporation, Redmond, WA).

The census generated a list of 21,496 persons. We eliminated cases with no data and those with no lower-limb lymphedema, and 17,036 respondents remained in the dataset.

Gender and age distribution of morbidity. From the Khurda morbidity census data we generated the gender and age distribution of persons with lymphedema. As shown in Supplemental Table 1, there is only a small gender difference in the number of ADLA episodes, as others have found[32,33] (for contrary findings, see Refs. 34, 35).

As shown in Supplemental Table 2, there were only small differences in lymphedema stage between females and males. The "average stage" was slightly higher for females than males (2.14 versus 2.06). (Average stage is in quotation marks in the previous sentence since stage is an ordinal, not a cardinal, variable.) Men were more likely to be in stage 1 lymphedema than women, and women were more likely to be in stages 2 and 3 than men. There was more gender balance at higher stages.

Supplemental Table 1
Number of ADLA episodes in previous year by gender in Khurda census, 2005

Gender	Number of respondents	Percentage of females and males having ADLA episodes in 1 year					Average number of ADLA episodes
		0 episode	1 episode	2 episodes	3 episodes	Total	
Female	8,746	16.8	68.8	8.1	6.2	100.0	1.04
Male	8,290	15.3	69.8	8.4	6.6	100.0	1.06
Total	17,036	16.1	69.3	8.2	6.4	100.0	1.05

ADLA = acute dermatolymphangioadenitis.

Start date. CASA scale-up of the lymphedema management program was carried out between mid-2007 and mid-2010, with the largest number of people enrolled in 2008–2009.[36] We took mid-2008 to mid-2009 as our start date and calculated real wages and out-of-pocket medical costs from that time.

Age cohorts. Age was reported as ending in 0 or 5 for 72% of respondents, rather than the approximately 20% that one would expect. Accordingly, we grouped people in 5-year cohorts: 18- to 22-year-olds belong to the 20-year cohort, 23- to 27-year-olds belong to the 25-year cohort, and so on.

Age limits. The Khurda dataset included persons aged 3 to 99 years. We omitted the 18 children from 3 to 7 years of age because of small numbers, uncertainty of the cause of their reported lymphedema and ADLA, and the expected effect of cleared infection from their having experienced multiple rounds of MDA at a very young age.[18] Ultrasonographic and histological evidence shows lymphatic vessel damage in infected children long before the typical age at which lymphedema may become evident, generally around the age of 20 years.[37,38] In India and other endemic countries, there are reported cases of children in filaria-endemic zones with lymphedema thought to be of filarial origin.[39] Among respondents in the dataset, 1.3% were under the age of 18 years.

We assumed that those in the 10- and 15-year cohorts would incur out-of-pocket costs for lymphedema and ADLA from the beginning of the period calculated. The Indian Bureau of Labour does not include children under 15 years in the labor force, and we do not include these two cohorts in the lost earnings calculations until they reach the 20-year cohort (minimum age of 18 years). There is no maximum age listed for labor force participation in the Indian government labor statistics, but we calculated only up to the 70-year cohort (with 72 years as the maximum age) because of the sharp decline in respondents older than 72 years, Indian life expectancy of 66 years (http://data.worldbank.org/indicator/SP.DYN.LE00.IN), and decreased labor force participation. We calculated economic cost for the 15,853 people aged 8–72 years.

Women's labor force participation. We assigned economic loss to the disability of patients whether or not they were in the paid labor force. Specifically, women whose only or primary occupation is to prepare food, care for children or the sick and elderly, and other domestic activities are necessary for the household and the community to function as economic entities. We assign the same number of work days lost to men and women and the same average wage to each work day lost by either gender.

Out-of-pocket costs of medical treatment of ADLA per episode. We calibrated our model based on a study in Khurda District in 2000–2001 by Babu and Nayak (Table 1)[40] who reported that ADLA patients who sought medical care paid ₹ (Indian rupees) 92.3 on average (arithmetic mean) per episode of ADLA, which was equal to US$2.04. (For this amount and all other costs reported in rupees, we convert to U.S. dollars for the appropriate dates using the OANDA online currency converter at http://www.oanda.com/currency/historical-rates/).

Babu and Nayak's estimated cost of US$2.04 per episode in Khurda is somewhat higher than reported in earlier studies in other regions of India. Nanda and Krishnamoorthy (p. 57)[41] reported per-episode treatment costs averaging US$0.46. From the data given in Table 2 in the work of Ramaiah and others,[42] one can calculate the arithmetic mean of per-episode spending on ADLA, which is US$0.61. Other studies provide estimates of annual spending on ADLA, not per-episode costs. Krishnamoorthy[43] reported spending of US$0.69 to US$3.00. In Tamil Nadu, Ramaiah and others (Table 2)[44] estimated the number of ADLA episodes and total spending by patients on ADLA in India. From those estimates, one can calculate the annual per-person (but not per-episode) cost of ADLA: US$1.01 for men and US$0.77 for women. We do not consider reported costs of treating ADLA in other countries.[45–47]

Annual out-of-pocket costs of medical treatment of lymphedema patients. We calibrated our model based on a study in Khurda District by Babu and others (p. 34)[48] who found that the average male lymphedema patient spent ₹ 576 annually and the average female spent ₹ 425, the weighted

Supplemental Table 2
Stage of lymphedema by gender in Khurda census, 2005

Gender	Number of respondents	Percentage of females and males at each stage of lymphedema								Average stage
		Stage of lymphedema								
		1	2	3	4	5	6	7	Total	
Female	8,746	34.6	31.3	24.8	6.3	1.7	0.9	0.5	100.0	2.14
Male	8,290	43.4	25.7	19.6	7.2	1.8	1.5	0.9	100.0	2.06
Total	17,036	38.9	28.6	22.3	6.7	1.7	1.2	0.7	100.0	2.10

average of which was ₹ 478, or US$10.96. The reported averages are geometric means, which are lower than arithmetic means. Even though the geometric mean for some purposes may offer a better sense of the central tendency of a highly skewed distribution, the appropriate mean for our modeling is the arithmetic mean since we estimated spending on medical services by all lymphedema patients. The estimate based on geometric mean thus understates the cost of treatment and generates a more conservative estimate of the benefits of the intervention.

Estimates of treatment costs for chronic lymphedema in other locales and at other times are lower. Nanda and Krishnamoorthy (p. 57)[41] reported per-visit treatment costs in south India averaging US$0.56 but did not report annual number of visits. Ramaiah and others (p. 21)[49] found that the geometric mean of annual spending on medical care for lymphedema patients in south India in 1993–1994 was ₹ 76 or US$2.42. Ramaiah and others (Table 2)[44] found annual treatment costs of chronic lymphedema (and hydrocele) to be US 2.25 and US$1.88 for men and women, respectively, in India as a whole.

Annual real increase in out-of-pocket treatment costs. We assumed that the demand for medical services will grow as household income rises (demand is income elastic). Moreover, since labor is a chief input into medical care provision, the cost of medical services will also rise as real wages rise. We assumed that the rate of growth in real (adjusted for inflation) expenditure on medical services will be the same as the rate of growth in real wages.

Average daily wage. The wage is useful for estimating the impact on individuals and their families, as well as the impact of their reduced spending in the community. It would also be desirable to measure the lost productivity for society that morbidity and disability entail. For that purpose, the wage is an imperfect proxy since it may not accurately measure the contribution of workers to output, nor the loss of their contribution to society. We estimated the average daily wage of unskilled, mostly agricultural workers, who comprise almost all (98%)[36] of the people in our dataset and are also representative of the population across India affected by lymphedema and ADLA. We calculated the average daily wage in rural occupations in Odisha State during the crop year July 2008 through June 2009. To determine the average daily wage, we used data from *Wage Rates in Rural India 2008–2009* (Tables 3a–14b),[50] which gives average wages for men and women for every month reported separately for ploughing, sowing, weeding, transplanting, harvesting, winnowing, threshing, picking, herding, and unskilled nonagricultural labor. We took the unweighted (the number of workers in each occupation is not given) average of wages in those 10 occupations averaged over 12 months of the crop year. Finally, we averaged men's and women's wages, weighted by their relative presence in the Khurda census, which was 48.7% male.

Annual increase in real daily wage. Between 2006–2007 and 2011–2012, real GDP in India grew 7.9% annually and real rural wages grew 6.8% (p. 12)[51] (see also Refs. 52–54). Since 2003, real GDP growth exceeded 9% in 4 years and 7% in 8 years. The OECD projects 6.1% growth of real GDP in India in 2016, gradually trending downward to about 4.6% annually by 2040.[55] Between 2007 and 2011, Odisha had the most rapid growth in rural wages in India (p. 189).[54] Indian economists report the following forces pushing up rural wages: rapid growth in GDP, a shrinking share of agriculture in GDP, growth of nonagricultural economic activity in rural areas, and rapid urbanization due to rapid growth in urban employment, causing a growing gap between urban and rural wages and fostering rural labor shortages, growing mechanization, and growing demand for skilled agricultural workers. Other factors include increasing integration of labor markets across states and national and state government policies that promote growth in rural wage rates, especially the Mahatma Gandhi National Rural Employment Guarantee Act, which has boosted rural wages since 2006 by guaranteeing employment of at least 100 days/year for low-income workers.[51–54] All of that suggests that rural wage growth could easily reach 4% annually over the coming decades. Accordingly, we set our baseline prediction of real rural wage growth at 4%. The recent rapid growth in real rural wages may be a harbinger of even more rapid growth in wages over the coming decades. Our sensitivity analysis, therefore, explores the possibility that real rural wages in India could grow as rapidly as 5% annually over the coming decades.

On the other hand, real farm wages in India grew at an average annual rate of only 2.9% from 1990 to 2012.[51] Furthermore, between 2000 and 2006, real farm wages fell by nearly 2% annually.[51] Despite rapid wage growth in 2006–2011, both Indian GDP growth and real rural wage growth slowed sharply in 2012.[56] Persistently weak economic performance in Europe and the United States is currently a drag on the Indian economy that could continue long into the future. All of this could suggest that the baseline assumption of 4% annual growth in real wages is too optimistic, so the sensitivity analysis shows the effect on the economic cost of lymphedema and ADLA if real rural wages continue the 2.9% pace set between 1990 and 2012.

Days of work lost per year by people with chronic lymphedema. Lymphedema can reduce one's ability to engage in productive work, and we measured that as days of work lost. Several studies record reduced hours of labor per day, reduced physical output, absenteeism, and coping by performing less strenuous and lower paid jobs.[44,48,49,57–59] We estimated a composite figure to represent all of the ways that morbidity reduces productivity and earnings. Lower productivity that reduces the wage rate is treated as a partial reduction in days worked and is subsumed into our measure of days of work lost. To determine the days lost, first we found the number of days a usually occupied rural laborer in Odisha could expect to work in a year. We relied on data for Odisha State from Table 4.1.1.1.1, Average Annual Number of Days Not Worked by Usually Occupied Men Belonging to All Classes of Rural Labour Households, from the Rural Labour Enquiry (61st Round of N.S.S.) 2004–05, Report on Employment & Unemployment of Rural Labour Households (Main Report), published by the Labour Bureau of the Government of India.[60] The report indicates that men in rural areas of Odisha State did not work an average of 76 days during the crop year 2005–2006 because no work was available or they were not available for work (since they were, for example, students, pensioners, or disabled). Consequently, usually occupied men in rural Odisha worked an average of 289 days per year. Many women are engaged fully or partially in unpaid work. As indicated above, we assigned women the same number of potential work days as men since their work in the household is essential to the household and community economy.

Supplemental Table 3
Sensitivity testing: variations in wages, prices, and work time loss

	Assumptions subject to sensitivity testing				
	Annual increase in real wages and real out-of-pocket spending on medical services			Loss of work time due to lymphedema	
	Baseline 4.0%	2.9%	5.0%	Baseline* work time loss (11.2%)	Higher† work time loss (15.2%)
Economic cost without lymphedema management (in millions of US$)	47.4	39.2	57.0	47.4	58.8
Economic cost with lymphedema management (in millions of US$)	21.3	19.2	23.5	21.3	27.1
Reduction in economic cost with lymphedema management (in millions of US$)	26.1	20.0	33.6	26.1	31.7
Reduction in economic cost per person with lymphedema management (in US$)	1,648	1,263	2,119	1,648	1,998

*Stages 1–2 = 0%; stage 3 = 20%; stage 4 = 50%; stages 5–7 = 100%.
†Stage 1 = 0%; stage 2 = 10%; stage 3 = 25%; stage 4 = 50%; stages 5–7 = 100%.

Although there are numerous estimates of lost work time due to chronic lymphedema, none reports the degree of disability by stage of lymphedema, measured either as hours or days of work (and thus lost wages) or reduced wage rates. For the baseline calculations, we assumed that lymphedema patients in stages 1 and 2 do not lose any work time due to symptoms of lymphedema. We assume that lymphedema patients in stages 5–7 cannot work, losing 289 days of productive activity annually because of chronic lymphedema. We further assumed that patients with stage 3 lymphedema experience a 20% reduction in work time and patients with stage 4 lymphedema experience a 50% reduction in work time as a composite estimate of reduced hours, reduced intensity of work, lower pay grade, and absenteeism. The Khurda census allows us to compute the number of respondents in each age cohort at each stage of lymphedema. We multiplied that number by the average daily wage and summed over all cohorts and all stages to determine the economic cost of chronic lymphedema from lost earnings.

Our assumptions about degree of disability at each stage of lymphedema were quite conservative. They produce an average of 32 lost work days annually for persons with chronic lymphedema in stages 1–7. That is much lower than the loss reported in the work of Ramaiah and others, who found that in India as a whole those with lymphedema or hydrocele lost 51 days of work annually (Table 3).[44] Several studies show that lost work days for men with lymphedema or hydrocele are about the same, so Ramaiah and others' estimate is an appropriate indicator of lost work time for lymphedema.[48,49,57,58]

Our assumptions about lost work time at different stages of lymphedema result in an 11.2% reduction in work time, which is substantially lower than other studies have found. Ramaiah and others (Table 4)[49] indicate that lymphedema patients worked 15.2% less than controls. Ramaiah and others (Tables 3 and 4)[58] reported that those with chronic lymphedema worked 13.7% less than controls in paid work and 13.0% less in unpaid domestic work. Babu and others (Table 3)[48] found males with lymphedema worked 15.4% less than controls and women worked 23.5% less. Babu and others (p. 714)[57] measured a 20.2% drop in earnings for weavers with lymphedema (8.0% from lower wages and 12.2% from fewer hours worked). Similarly, Ramu and others (p. 670)[59] found that male weavers with lymphedema produced 27.4% less cloth than those with no lymphedema. Our baseline assumption about productivity loss from lymphedema is thus lower than any other published estimate. Accordingly, the sensitivity analysis determines the effect of assuming greater disability among those with lymphedema than in the baseline analysis, with work time loss as follows: stage 1 = 0%, stage 2 = 10%, stage 3 = 25%, stage 4 = 50%, stages 5–7 = 100%. These assumptions produce a 15.2% average work time loss for all persons in the Khurda census dataset (or 44 lost work days annually), which is just below the mean of what other studies have found.

Average length of ADLA episodes/days of work lost from ADLA. We calibrated our model based on the work of Babu and Nayak (p. 1104)[40] and Babu and others (Table 1)[61] who reported on a study in Khurda District that finds a mean duration of ADLA episodes of approximately 4 days (3.93 days). The length of ADLA episodes in adults has been reported in numerous other studies.[34,35,42,44,58,62–68] The range of reported average duration of an ADLA episode is 3–8.6 days with a mean duration of 4.86 days.

Discount rate. We used an annual discount rate of 3%, which is the standard rate used for analyzing health interventions.[69]

Results of sensitivity analysis. We addressed uncertainty about appropriate values for some of the parameters in our modeling with sensitivity analysis. We projected a 4% annual growth in real wages and a commensurate growth in out-of-pocket spending for medical services associated with chronic lymphedema and ADLA. As shown in Supplemental Table 3, reducing predicted growth in the real wage to 2.9% (the average rate between 1990 and 2012) reduced the per-person reduction in the economic cost of lymphedema and ADLA by 23%, from US$1,648 to US$1,263. In the last decade, rural real wages in India and in Odisha have grown far faster than 2.9%, suggesting that a 5% annual growth in real wages is within the realm of possibility. Modeling a 5% real wage growth raised the per-person payoff of implementing a lymphedema management program by 29%, to US$2,119.

Our assumptions about the degree of disability caused by lymphedema at different stages produced a loss in work time of just over 11%. Other studies on the burdens of lymphedema have found a loss of work time between 13.0% and 23.5% with a mean of 15.6%[48,49,58] (see also Refs. 57,59). Our 11.2% worktime loss may be appropriate, given that our data were based on a census that sought to find every person in the target districts with lower-limb lymphedema. Studies that recruit participants who come to clinics as patients, for example, may overrepresent individuals at higher stages of lymphedema since those with early or mild lymphedema may not seek health services. In case our estimates were too

conservative, we reestimated our model to produce a 15.2% total loss in work time, just below the mean work time loss found in other research. Doing so raised the per-person reduction in economic burden of lymphedema and ADLA by 21%, from US$1,648 to US$1,998, at 4% rate of growth of real wages and costs and 3% discount rate. The comparison of results with baseline parameters and sensitivity analysis is shown in Supplemental Table 3.

SUPPLEMENTAL REFERENCES

1. WHO, 2004. Lymphatic filariasis: progress of disability prevention activities. *Wkly Epidemiol Rec 79:* 417–424.
2. El-Nahas H, El-Shazly A, Abulhassan M, Nabih N, Mousa N, 2011. Impact of basic lymphedema management and antifilarial treatment on acute dermatolymphangioadenitis episodes and filarial antigenaemia. *J Glob Infect Dis 3:* 227–232.
3. Akogun OB, Badaki JA, 2011. Management of adenolymphangitis and lymphoedema due to lymphatic filariasis in resource-limited north-eastern Nigeria. *Acta Trop 120 (Suppl 1):* S69–S75.
4. Jullien P, Somé Jd A, Brantus P, Bougma RW, Bamba I, Kyelem D, 2011. Efficacy of home-based lymphoedema management in reducing acute attacks in subjects with lymphatic filariasis in Burkina Faso. *Acta Trop 1205:* 555–561.
5. Wijesinghe RS, Wickremasinghe AR, Ekanayake S, Perera MS, 2007. Efficacy of a limb-care regime in preventing acute adenolymphangitis in patients with lymphoedema caused by bancroftian filariasis, in Colombo, Sri Lanka. *Ann Trop Med Parasitol 101:* 487–497.
6. Aggithaya MG, Narahari SR, Vayalil S, Shefuvan M, Jacob NK, Sushma KV, 2013. Self care integrative treatment demonstrated in rural community setting improves health related quality of life of lymphatic filariasis patients in endemic villages. *Acta Trop 126:* 198–204.
7. Shenoy R, Kumaraswami V, Suma T, Rajan K, Radhakuttyamma G, 1999. A double-blind, placebo-controlled study of the efficacy of oral penicillin, diethylcarbamazine or local treatment of the affected limb in preventing acute adenolymphangitis in lymphoedema caused by Brugian filariasis. *Ann Trop Med Parasitol 93:* 367–377.
8. Shenoy R, Suma T, Rajan K, Kumaraswami V, 1999. Prevention of acute adenolymphangitis in Brugian filariasis: comparison of the efficacy of ivermectin and diethylcarbamazine, each combined with local treatment of the affected limb. *Ann Trop Med Parasitol 92:* 587–594.
9. Suma T, Shenoy R, Kumaraswami V, 2002. Efficacy and sustainability of a footcare programme in preventing acute attacks of adenolymphangitis in Brugian filariasis. *Trop Med Int Health 7:* 763–766.
10. Joseph A, Mony P, Prasad M, John S, Srikanth Mathai D, 2004. The efficacies of affected-limb care with penicillin diethylcarbamazine, the combination of both drugs or antibiotic ointment, in the prevention of acute adenolymphangitis during bancroftian filariasis. *Ann Trop Med Parasitol 98:* 685–696.
11. Narahari SR, Bose KS, Aggithaya MG, Swamy GK, Ryan TJ, Unnikrishnan B, Washington RG, Rao BP, Rajagopala S, Manjula K, Vandana U, Sreemol TA, Rojith M, Salimani SY, Shefuvan M, 2013. Community level morbidity control of lymphoedema using self care and integrative treatment in two lymphatic filariasis endemic districts of south India: a non randomized interventional study. *Trans R Soc Trop Med Hyg 107:* 566–577.
12. Ryan TJ, Narahari SR, 2012. Reporting an alliance using an integrative approach to the management of lymphedema in India. *Int J Low Extrem Wounds 11:* 5–9.
13. Addiss DG, Louis-Charles J, Roberts J, Leconte F, Wendt JM, Milord MD, Lammie PJ, Dreyer G, 2010. Feasibility and effectiveness of basic lymphedema management in Leogane, Haiti, an area endemic for bancroftian filariasis. *PLoS Negl Trop Dis 4:* e668.
14. Addiss DG, Michel MC, Michelus A, Radday J, Billhimer W, Louis-Charles J, Roberts JM, Kramp K, Dahl BA, Keswick B, 2011. Evaluation of antibacterial soap in the management of lymphoedema in Leogane, Haiti. *Trans R Soc Trop Med Hyg 105:* 58–60.
15. Mathieu E, Dorkenoo AM, Datagni M, Cantey PT, Morgah K, Harvey K, Ziperstein J, Drexler N, Chapleau G, Sodahlon Y, 2013. It is possible: availability of lymphedema case management in each health facility in Togo: program description, evaluation, and lessons learned. *Am J Trop Med Hyg 89:* 16–22.
16. Stocks ME, Freeman MC, Addiss DG, 2015. The effect of hygiene-based lymphedema management in lymphatic filariasis-endemic areas: a systematic review and meta-analysis. *PLoS Negl Trop Dis 9:* 1–19.
17. Bockarie MJ, Tisch DJ, Kastens W, Alexander ND, Dimber Z, Bockarie F, Ibam E, Alpers MP, Kazura JW, 2002. Mass treatment to eliminate filariasis in Papua New Guinea. *N Engl J Med 347:* 1841–1848.
18. Ramaiah KD, Ottesen EA, 2014. Progress and impact of 13 years of the Global Programme to Eliminate Lymphatic Filariasis on reducing the burden of filarial disease. *PLoS Negl Trop Dis 8:* e3319.
19. Mackenzie CD, Lazarus WM, Mwakitalu ME, Mwingira U, Malecela MN, 2009. Lymphatic filariasis: patients and the global elimination programme. *Ann Trop Med Parasitol 103 (Suppl 1):* S41–S51.
20. Meyrowitsch DW, Simonsen PE, Makunde WH, 1996. Mass DEC chemotherapy for control of bancroftian filariasis: comparative efficacy of four strategies two years after start of treatment. *Trans R Soc Trop Med Hyg 90:* 423–428.
21. Partono F, 1985. Treatment of elephantiasis in a community with timorian filariasis. *Trans R Soc Trop Med Hyg 79:* 44–46.
22. Shenoy RK, Suma TK, Kumaraswami V, Rahmah N, Dhananjayan G, Padma S, 2009. Antifilarial drugs, in the doses employed in mass drug administrations by the Global Programme to Eliminate Lymphatic Filariasis, reverse lymphatic pathology in children with *Brugia malayi* infection. *Ann Trop Med Parasitol 103:* 235–247.
23. Tisch DJ, Alexander ND, Kiniboro B, Dagoro H, Siba PM, Bockarie MJ, Alpers MP, Kazura JW, 2011. Reduction in acute filariasis morbidity during a mass drug administration trial to eliminate lymphatic filariasis in Papua New Guinea. *PLoS Negl Trop Dis 5:* e1241.
24. Casley-Smith JR, Jamal S, Casley-Smith J, 1993. Reduction of filaritic lymphoedema and elephantiasis by 5,6 benzo-α-pyrone (coumarin), and the effects of diethylcarbamazine (DEC). *Ann Trop Med Parasitol 87:* 247–258.
25. Eddy BA, Blackstock AJ, Williamson JM, Addiss DG, Streit TG, Beau de Rochars VM, Fox LM, 2014. A longitudinal analysis of the effect of mass drug administration on acute inflammatory episodes and disease progression in lymphedema patients in Leogane, Haiti. *Am J Trop Med Hyg 90:* 80–88.
26. Malecela MN, Mwingira U, Mwakitalu ME, Kabali C, Michael E, Mackenzie CD, 2009. The sharp end—experiences from the Tanzanian programme for the elimination of lymphatic filariasis: notes from the end of the road. *Ann Trop Med Parasitol 103 (Suppl 1):* S53–S57.
27. Kerketta A, Babu B, Rath K, Jangid P, Nayak A, Kar S, 2005. A randomized clinical trial to compare the efficacy of three treatment regimens along with footcare in the morbidity management of filarial lymphoedema. *Trop Med Int Health 10:* 698–705.
28. Das L, Subramanyam Reddy G, Pani S, 2003. Some observations on the effect of Daflon (micronized purified flavonoid fraction of *Rutaceae aurantiae*) in bancroftian filarial lymphoedema. *Filaria J 12:* 5.
29. Dunyo S, Nkrumah F, Simonsen P, 2000. Single-dose treatment of *Wuchereria bancrofti* infections with ivermectin and albendazole alone or in combination: evaluation of the potential for control at 12 months after treatment. *Trans R Soc Trop Med Hyg 94:* 437–443.
30. Mues KE, Deming M, Kleinbaum DG, Budge PJ, Klein M, Leon JS, Prakash A, Rout J, Fox LM, 2014. Impact of a community-based lymphedema management program on episodes of adenolymphangitis (ADLA) and lymphedema progression—Odisha State, India. *PLoS Negl Trop Dis 8:* e3140.

31. Budge PJ, Little KM, Mues KE, Kennedy ED, Prakash A, Rout J, Fox LM, 2013. Impact of community-based lymphedema management on perceived disability among patients with lymphatic filariasis in Orissa State, India. *PLoS Negl Trop Dis 7:* e2100.
32. Pani SP, Krishnamoorthy K, Rao AS, Prathiba J, 1991. Clinical manifestations in Malayan filariasis infection with special reference to lymphoedema grading. *Indian J Med Res 91:* 200–207.
33. Pani S, Balakrishnan N, Srividya A, Bundy A, Grenfell B, 1991. Clinical epidemiology of bancroftian filariasis: effect of age and gender. *Trans R Soc Trop Med Hyg 85:* 260–264.
34. Gyapong JO, Gyapong M, Adjei S, 1996. The epidemiology of acute adenolymphangitis due to lymphatic filariasis in northern Ghana. *Am J Trop Med Hyg 54:* 591–595.
35. Ramaiah K, Ramu K, Vijay Kumar K, Guyatt H, 1996. Epidemiology of acute filarial episodes caused by *Wuchereria bancrofti* infection in two rural villages in Tamil Nadu, south India. *Trans R Soc Trop Med Hyg 90:* 639–643.
36. Fox LM, Rout J, Budge P, Prakash A, Michyari A, Little KM, 2011. *Quantifying the Economic Benefits of a Community-based Lymphedema Management Program—Orissa State, India*. American Society of Tropical Medicine and Hygiene Annual Meeting, Philadelphia, PA, December 4–8, 2011.
37. Fox LM, Furness BW, Haser JK, Brissau JM, Louis-Charles J, Wilson SF, Addiss DG, Lammie PJ, Beach MJ, 2005. Ultrasonographic examination of Haitian children with lymphatic filariasis: a longitudinal assessment in the context of antifilarial drug treatment. *Am J Trop Med Hyg 72:* 642–648.
38. Dreyer G, Figueredo-Silva J, Carvalho K, Amaral F, Ottesen E, 2001. Lymphatic filariasis in children: adenopathy and its evolution in two young girls. *Am J Trop Med Hyg 65:* 204–207.
39. Ramaiah KD, Vijay Kumar KN, 2000. Effect of lymphatic filariasis on school children. *Acta Trop 76:* 197–199.
40. Babu B, Nayak A, 2003. Treatment costs and work time loss due to episodic adenolymphangitis in lymphatic filariasis patients in rural communities of Orissa, India. *Trop Med Int Health 8:* 1102–1109.
41. Nanda B, Krishnamoorthy K, 2003. Treatment seeking behaviour and costs due to acute and chronic forms of lymphatic filariasis in urban areas in south India. *Trop Med Int Health 8:* 56–59.
42. Ramaiah KD, Ramu K, Guyatt H, Vijar Kumar KN, Pani SP, 1998. Direct and indirect costs of the acute form of lymphatic filariasis to households in rural areas of Tamil Nadu, south India. *Trop Med Int Health 3:* 108–115.
43. Krishnamoorthy K, 1999. Estimated costs of acute adenolymphangitis to patients with chronic manifestations of bancroftian filariasis in India. *Indian J Public Health 43:* 58–63.
44. Ramaiah KD, Das PK, Michael E, Guyatt H, 2000. The economic burden of lymphatic filariasis in India. *Parasitol Today 16:* 251–253.
45. Chandrasena T, Premaratna RR, de Silva N, 2004. Lymphoedema management knowledge and practices among patients attending filariasis morbidity clinics in Gampaha District, Sri Lanka. *Filaria J 3:* 6.
46. Gyapong JO, Gyapong M, Evans DB, Aikins MK, Adjei S, 1996. The economic burden of lymphatic filariasis in northern Ghana. *Ann Trop Med Parasitol 90:* 39–48.
47. Kron M, Walker E, Hernandez L, Torres E, Libranda-Ramirez B, 2000. Lymphatic filariasis in the Philippines. *Parasitol Today 16:* 329–333.
48. Babu B, Nayak A, Dahl K, Acharya A, Jangrid P, Mallick G, 2002. The economic loss due to treatment costs and work loss to individuals with chronic lymphatic filariasis in rural communities of Orissa, India. *Acta Trop 82:* 31–38.
49. Ramaiah K, Guyatt H, Ramu K, Vanamail P, Pani S, Das P, 1999. Treatment costs and loss of work time to individuals with chronic lymphatic filariasis in rural communities in south India. *Trop Med Int Health 4:* 19–25.
50. Labour Bureau, 2010. *Wage Rates in Rural India 2008–2009*. Shimla/Chandigarh, India: Ministry of Labour and Employment, Government of India.
51. Gulati A, Jain S, Satija N, 2013. *Rising Farm Wages in India: The 'Pull' and 'Push' Factors*. New Delhi, India: Commission for Agricultural Costs and Prices, Department of Agriculture and Cooperation, Ministry of Agriculture, 1–29.
52. Reddy AA, 2013. Trends in rural wage rates: whether India reached Lewis turning point? *Social Science Research Network*. DOI:10.2139/ssrn.2321491.
53. Chavan P, Bedamatta R, 2006. Trends in agricultural wages in India 1964-65 to 1999-2000. *Econ Polit Wkly 41:* 4041–4051.
54. Usami Y, 2012. Recent trends in wage rates in rural India: an update. *Rev Agrarian Stud 2:* 171–181.
55. OECD.Stat, 2015. *Economic Outlook No 95—May 2014—Long-Term Baseline Projections*. Available at: http://stats.oecd.org/Index.aspx?DataSetCode=EO95_LTB#. Accessed July 24, 2015.
56. Rukmini SM, Venu K, 2013. Dip in rural wage growth rate may dent UPA vote. *The Hindu*. New Delhi, India.
57. Babu B, Swain B, Rath K, 2006. Impact of chronic lymphatic filariasis on quantity and quality of productive work among weavers in an endemic village from India. *Trop Med Int Health 11:* 712–717.
58. Ramaiah KD, Radhamani MP, John KR, Evans DB, Guyatt H, Joseph A, 2000. The impact of lymphatic filariasis on labour inputs in southern India: results of a multi-site study. *Ann Trop Med Parasitol 94:* 353–364.
59. Ramu K, Ramaiah KD, Guyatt H, Evans D, 1996. Impact of lymphatic filariasis on the productivity of male weavers in a south Indian village. *Trans R Soc Trop Med Hyg 90:* 669–670.
60. Labour Bureau, ND. *Rural Labour Enquiry (61st Round of N.S.S.) 2004–05 Report on Employment & Unemployment of Rural Labour Households (Main Report)*. Shimla/Chandigarh, India: Ministry of Labour and Employment, Government of India.
61. Babu B, Nayak A, Dhal K, 2005. Epidemiology of episodic adenolymphangitis: a longitudinal prospective surveillance among a rural community endemic for bancroftian filariasis in coastal Orissa, India. *BMC Public Health 5:* 1–6.
62. Abidha SL, Das LK, Yuvraj J, Vijayalaxmi G, Pani SP, 2008. The plight of chronic filarial lymphoedema patients in choice of health care and health care providers in Pondicherry, India. *J Commun Dis 40:* 101–109.
63. Richard S, Mathieu E, Addiss D, Sodahlon Y, 2007. A survey of treatment practices and burden of lymphoedema in Togo. *Trans R Soc Trop Med Hyg 101:* 391–397.
64. Gasarasi D, Premji Z, Mujinja P, Mpembeni R, 2000. Acute adenolymphangitis due to bancroftian filariasis in Rufiji district, south east Tanzania. *Acta Trop 75:* 19–28.
65. Pani S, Yuvaraj J, Vanamail P, Dhanda V, Michael E, Grenfell B, Bundy D, 1995. Episodic adenolymphangitis and lymphoedema in patients with bancroftian filariasis. *Trans R Soc Trop Med Hyg 89:* 72–74.
66. Sabesan S, Krishnamoorthy K, Pani S, Panicker K, 1992. Mandays lost due to repeated attacks of lymphatic filariasis. *Trends Life Sci 7:* 5–7.
67. Rao C, Chandrasekharan A, Cherian C, 1982. Frequency and duration of acute filarial attacks in persons in *Brugia malayi* endemic community. *Indian J Med Res 75:* 813–815.
68. Ramaiah K, Kumar K, 2000. Effect of lymphatic filariasis on school children. *Acta Trop 76:* 197–199.
69. Corso PS, Haddix AC, 2003. Time effects. Haddix AC, Teutsch SM, Corso PS, eds. *Prevention Effectiveness: A Guide to Decision Analysis and Economic Evaluation*. New York, NY: Oxford University Press, 92–102.
